# Ethical and methodological issues in research with Sami experiencing disability

**DOI:** 10.3402/ijch.v75.31656

**Published:** 2016-07-06

**Authors:** Line Melbøe, Ketil Lenert Hansen, Bjørn-Eirik Johnsen, Gunn Elin Fedreheim, Tone Dinesen, Gunn-Tove Minde, Marit Rustad

**Affiliations:** Department of Social Education, University of Tromsø – The Arctic University of Norway, Harstad, Norway

**Keywords:** ethical and methodological issues, Sami, indigenous, health, disability, Norway

## Abstract

**Background:**

A study of disability among the indigenous Sami people in Norway presented a number of ethical and methodological challenges rarely addressed in the literature.

**Objectives:**

The main study was designed to examine and understand the everyday life, transitions between life stages and democratic participation of Norwegian Sami people experiencing disability. Hence, the purpose of this article is to increase the understanding of possible ethical and methodological issues in research within this field. The article describes and discusses ethical and methodological issues that arose when conducting our study and identifies some strategies for addressing issues like these.

**Methods:**

The ethical and methodological issues addressed in the article are based on a qualitative study among indigenous Norwegian Sami people experiencing disability. The data in this study were collected through 31 semi-structured in-depth interviews with altogether 24 Sami people experiencing disability and 13 next of kin of Sami people experiencing disability (8 mothers, 2 fathers, 2 sister and 1 guardian).

**Findings and discussion:**

The researchers identified 4 main areas of ethical and methodological issues. We present these issues chronologically as they emerged in the research process: 1) concept of knowledge when designing the study, 2) gaining access, 3) data collection and 4) analysis and accountability.

**Conclusion:**

The knowledge generated from this study has the potential to benefit future health research, specifically of Norwegian Sami people experiencing disability, as well as health research concerning indigenous people in general, providing scientific-based insight into important ethical and methodological issues in research with indigenous people experiencing disability.

The goal of this article is to describe and discuss ethical and methodological issues emerging in research with Sami people experiencing disability.

The Sami people are estimated to comprise between 60,000 and 110,000 individuals. The Sami are the indigenous people of *Sápmi*, a territory comprising parts of Arctic Norway, Sweden, Finland and Russia (1). Approximately, 70% of the Sami population lives in the Norwegian part of Sápmi. In recent decades, there has been considerable migration from traditional Sami municipalities to urban areas, implying a significant Sami (or multiethnic) population living in Norwegian towns and cities (2). Today, the Sami are represented in practically all the modern professions and trades; a majority of the Sami population has adopted the Western lifestyle (modern professions and food habits). Only small groups are still holding on to traditional ways of life (based on fishing, hunting and reindeer herding) (3–5). The Sami people in Norway have a close history of discrimination, that is, being forcibly discouraged from practicing their culture and language (6). Due to the more recent revitalization and integration of Sami culture and identity, the Sami have progressed from being strongly stigmatized to being generally treated as equals (7). According to the Sami Act of 1987, § 3–5, the Sami in Norway have a legal right to receive equitable health and social services adapted to Sami language and culture (8). In Norway, the delivery of primary healthcare and social services is the responsibility of municipalities, whereas “specialized health services” (including general and psychiatric hospitals, ambulances, substance abuse treatment and patient transportation) are provided by regional health authorities.

In this study, disability is understood according to the Nordic relational model, describing disability as a mismatch between the individual and the environment. The disability occurs both due to individual differences and because the environment is not adapted to accommodate the range of people. For example, a person using a wheelchair is disabled if stairs is the only option to get to the second floor, but not if there is an elevator (9). Impairment is often defined as the functional limitation within the individual caused by physical, mental or sensory impairment (10). Consequently, disability can be understood as the result of negative interactions taking place between a person with an impairment and her or his social environment. During the last two decades in Norway, there has been increasing research on disability in general (11,12). However, according to White Paper no. 45 (13), there is a lack of knowledge regarding the situation of disabled Sami people. This is especially true when it comes to physical and cognitive impairments (14).

Earlier research has caused strain and contributed to further stigmatization when it comes to the Sami (15) and people experiencing disability (16). Being Sami *and* disabled puts this group in an extra vulnerable position. Therefore, research with indigenous people like the Sami (15), and with the disabled (17), requires extra ethical awareness from the researcher. In recent years, many indigenous communities around the world, policy makers and researchers have criticized the academic community for not being aware of the specific challenges indigenous communities have faced and still are facing with regard to developing indigenous methodologies in research. One result of the decades of discourse in indigenous communities is the development in many Western countries of indigenously sensitive ethical research guidelines (18–20). For example, in Australia, Canada and New Zealand, there is an understanding of the protection of indigenous communities as well as individuals, where the participation of indigenous communities in research is an integral part of the indigenously sensitive ethical research guidelines. However, this is not the case in Norway. Where it is up to the individual researcher or research institution to decide whether and how to involve the indigenous (Sami) community perspective in their research projects (21). However, in 1997, the Sami Parliament in Norway reached a unanimous decision that ethical guidelines for Sami research had to be drawn up. However, such guidelines are still to be created (22).

## Objective

This article describes and discusses ethical and methodological issues that arose when conducting our study regarding Norwegian Sami people experiencing disability and suggests some strategies to address these issues.

We will now briefly describe the study which the article draws upon. Thereafter, we present our findings and discuss these consecutively. Finally, we present our conclusion.

## The study

This article draws on data from a research project that aimed to explore the everyday life, transitions between life stages and democratic participation of Sami people experiencing disability in Norway. This is a 2-year project funded by the Nordic Welfare Centre and Harstad University College.^1^ The project involves 31 qualitative interviews with 24 Sami people experiencing disability and 13 of next of kin of Sami people experiencing disability (8 mothers, 2 fathers, 2 sister and 1 guardian). The next of kin took part in the interviews either as support or as informants. This was either because of the young age (under 18 years old) of the informants or because the disabled person had trouble with answering the questions themselves. In general, the participants received an information letter in Norwegian and Sami and were asked to consent if they wanted to participate. When it comes to Sami people experiencing cognitive impairments as intellectual disability, we sent both an ordinary and an easy-read version of an information letter to the person's guardian or next of kin. In the information letter to the next of kin, we specified that valid consent implied that the person could possess sufficient information, understand the information given and be able to understand the implications of their consent (23). The guardian or next of kin then consented for those (by them) assessed as not being able to give an informed consent themselves. Next of kin or guardian then presented an easy read version of the information letter to the persons with intellectual disability, so that they themselves could approve whether to participate or not. Some of the next of kin decided that it was better to interview them than their brother or sister, since they did not have any or very little verbal language.

The 10 girls/women and 21 boys/men participating represented a range of disabilities, having sensory, physical or cognitive impairments. Both children, youth, grown-ups and the elderly took part in the study. The interviews were semi-structured but inspired by storytelling as we also urged the informants to talk more freely about their personal experiences and thoughts on being Sami experiencing a disability. The informants chose whether to be interviewed in Norwegian or in any of the 3 official Sami languages.

## Findings and discussion

### Concept of knowledge when designing the study

#### Awareness of Sami culture and traditional Sami knowledge influenced the design

Our first challenge was how to design a study that safeguarded traditional Sami knowledge. There is increasing international emphasis on preserving the traditional knowledge and social values of indigenous people (24), such as those of the Sami (25).

Based on research group members’ earlier experience, and on indigenous (22,26) and Sami epistemology and methodology literature (27–29), we were aware of the gap between the traditional Sami concept of knowledge and the Western scientific concept of knowledge.

Accordingly, an important issue when planning our research project was how to handle the difference between indigenous and Western research paradigms. Hence, we started the project by attending a seminar on Sami history and traditional knowledge. Methodologically, we found storytelling as a possible way to build a bridge between the Sami and Western concept of knowledge. Traditional Sami knowledge is often orally transmitted knowledge, connected to the belonging and participation in a specific cultural and social context (30). Storytelling is an ancient practice that has been used by indigenous cultures for thousands of years (31), which preserve and promote indigenous wisdom, celebrate myriad stories and lived experiences (32) and teach traditional ecological knowledge needed for survival in Sápmi homeland. Storytelling has a strong foothold inside Sami culture (33). Facilitating for storytelling at the interviews, we experienced that the participants spoke more freely about their experiences because storytelling provides a strong foundation for sharing life lessons and experiences, which reflect a *within-Sami-culture* view, drawing directly from personal stories and experiences (32,33). Still, it might be an ethical paradox that we asked the informants for stories associated with two possible stigmas, attached to being Sami and having an impairment, which therefore could be demanding to present. On the other hand, we experienced that our informants when telling stories spoke quite freely. A methodological implication was that these stories could not be compared, but instead served as background information related to Sami culture and context. This might be because it felt liberating to bring forward their experiences in a non-judgmental environment. We opened up for stories at the end of each interview, when we had got to know about each other. And we stressed that this was a possibility for them to choose what to focus on. Getting confirmation that they have been treated unacceptably, and at the same time contribute to limit this sort of mistreatment of others in the future (34).

#### Participation as a ground principle

Moreover, when it comes to research both the Sami (30) and people experiencing disability (35) have a history of having outsiders perspective imposed upon them, being researched by others without taking an active part in the research themselves. Hence, we based our choice of design upon the ethical principles of research involving indigenous peoples (24,36) and the agent perspective from modern disability research (37,38). Research represents knowledge, and knowledge is power. Hence, doing research with a minority people is about transferring control with research. This transfer of control and power becomes a part of gaining control with own living conditions, and secure that research draws upon local traditions, values and language.

There is now a growing recognition also by outsiders of the value and importance of involving both indigenous (24,36) and people experiencing disability (39) in the whole research process. This recognition represents a step away from what has long been recognized as a paternalistic approach by indigenous communities themselves. This is the primary source of information for formulating pertinent and essential research issues, which is in line with the British slogan “Nothing About Us Without Us,” used in disability studies where people experiencing disability argue that they alone can be the source for relevant research issues concerning their own lives (37). Hence, ethically, we made a design based on the researchers’ understanding of disabled Sami people as subjects and experts on their own lives.

However, involving the participants in all parts of the project is both time consuming and costly. Our project had a time limit and limited funding. This, unfortunately, prevented us from involving the Sami people experiencing disability as actively as desirable in all parts of the research process; for example, they were not involved in the preparation of the research questions. However, quite early we established a reference group that was involved in the rest of the research process. The group comprised members with a strong involvement in promoting the Sami communities and/or disability questions. The members represented both Sami and disability organizations, higher education and research, and health and social workers in Sápmi.

### Gaining access

#### Ask for collective consent

Research is very much a situated activity. To gain access to the Sami population, there were two contextual aspects we especially knew we had to take into consideration. First, the harsh assimilation process the Sami people were exposed to (30), being denied their own language and culture (40). Second, the negative experiences indigenous people have had with past research (41). For example, research that has disempowered communities, imposed stereotypes that reinforce internalized racism and benefitted the researchers’ careers but not provided anything in return to the communities struggling with health disparities (26). In the mid-1850s, a novel branch of science – physical anthropology – reached Scandinavia. Through the identification of “typical” Sami and Nordic racial traits, primarily the shape of the skull, it would be possible to empirically determine and trace which race first inhabited Europe's far north. A number of physical characteristics were associated with the measurement of skulls. The partitioning doubled as an “evolutionary scale” and the theories predicted the blonde “long-skulls” (the Nordic race) to be the superior product of evolution both in the bodily and spiritual sense. The Sami, on the other hand, belonged to the “short-skulls” and were described by the researcher Halvdan Bryn as being of a lesser and lower race that did not have a future. He writes “despite having lived in the immediate vicinity of more highly cultured races, they [the Sami] never arrived at any form of higher culture” (42). Some of the information was collected from living individuals; other measurements were conducted on skeletons from Christian and pre-Christian burial sites. Often, such excavations were performed in a manner which the Sami considered highly offensive and degrading (43). These studies are part of the tradition of craniometry science established by Samuel George Morton in America in the 1830s. He believed that brain size was linked to intelligence and used measuring of the interior cranial capacity as a scientific technique to rank human races (44). Scientific racism is the term used to describe this sort of studies where scientific techniques are used to support the belief of some racial categories being superior to others. We as researchers were aware of Sami people's negative experiences with skull studies. When presenting the project, we stressed our responsibility to ensure that the research did not impose further negative perceptions about them, hence separating us from scientific racism.

Due to negative experiences like these (6,45–47), we found it important to ask the Sami society about what Myrvoll (48) calls “collective consent” to conduct our research project. This was in respect for their right to control the knowledge production about themselves in the disability field. We therefore visited the Sami Parliament, presented the project to representatives and got their approval. Useful input from the representatives were taken into account; for example, the Parliament stressed the importance of having a sample including all parts of Norwegian Sàpmi, as much earlier research was conducted mainly with participants from the northern part. We acted on the advice from the Sami Parliament and made a strategic sample including individuals from both the Lule-, Southern- and Nothern Sami areas, and also some Sami living outside these areas. The collective consent from Sami parliament does, however, not absolve us as researchers from the need of obtaining informed consent from the participants. Hence, in addition to the collective consent, the Sami persons experiencing disability and/or their parent or guardian also had to consent. In 1997, the Sami Parliament in Norway reached a unanimous decision that ethical guidelines for Sami research had to be drawn up. Such guidelines are, however, still to be created (21). Hence, today Sami indigenous research in Norway is in the situation that the concept of the participation of indigenous communities in research is not an integral part of the Norwegian ethical guidelines (21).

#### Difficulties in identifying participants and making contact

Admitting to being Sami and having an impairment can be a sensitive matter. Except for the Sami Parliament's electoral roll (that is non-accessible for researchers), there is no public register of Sami people (49). Due to assimilation policies, many Sami have abandoned their Sami identity and avoid reporting Sami ethnicity (50). When recruiting participants, we therefore only wanted to request individuals who defined themselves as being Sami and disabled. Furthermore, we did not have permission to make direct contact with possible participants, but used health or social services to assist with recruitment. Based on an assumption that local public health and social services probably had a certain overview of who of the inhabitants was Sami and had an impairment, they were the first ones who were asked to pass on our request of participation in the project. We had our information letter translated, and sent our requests of participation in the three Sami languages and in the Norwegian language. Furthermore, we provided an easy-read version for those having learning difficulties and trouble with reading. However, a time-consuming process with a lot of phone calls and requests by the post resulted in only a few positive answers. There are probably several reasons for this low response. Among the responses, some reported the wording in the information sheet as alienating and too “professional”; for example, using phrases like “disability,” which made some individuals unsure whether or not they were potential participators. Others reacted to single words in the Sami language information sheet, finding them offensive (even though we had used official Sami language centres for translation). Because of this feedback, our information sheet was revised and a new version was written in a language more in line with Sami traditions. For example, instead of using the word disabled, we used phrases like “health-related challenges” connected to eyesight, hearing, movement, etc.

We also received comments from individuals offended by our request of participation in our study; for example, one person wrote “I am not interested, and do not want to receive any inquiries about any Sami research in the future.” This comment might be understood as an expression of shame in relation to Sami ethnicity because of the assimilation process in Norway or might also mean that people are tired of being researched, feel disenfranchised from the research process or do not feel there is any benefit from participating.

#### The best way to recruit is through formal and informal Sami networks

We decided to change our recruiting strategy and started contacting different formal and informal Sami networks, asking them to spread information and request about participation in the project. Sami networks included Sami Centres working with Sami language and culture, Sami political parties, Sami organizations, some of the researcher's personal networks, etc. Since we knew that the members of these networks identified themselves as Sami, we hoped to avoid offending any more individuals by ascribing them a Sami identity they did not agree with. One of the strategies to get in touch with the Sami networks was to organize meetings at official Sami Centres working with Sami language and culture. Here, we presented and discussed the project, got feedback and recruited participants. Furthermore, we asked newspapers located in the Sàpmi to present the project and our request of participants, and recruited more participants through these newspaper reports. In parallel, we also contacted our individual networks, especially the researchers with a Sami background, and recruited some participants. The result was that we got much faster access to relevant participants for the study.

### Data collection

#### Mismatch of concepts might be a challenge

Some participants chose the Sami language when interviewed. Since most of the researchers in our group do not speak or understand Sami, the interviews conducted in Sami were translated into Norwegian. However, some meanings might be lost or changed in translation (22). One of our informants stated that even though he understood Norwegian very well, he preferred Sami. This is because he was much more familiar with Sami, and how to get the right nuances to the fore. For example, there is no equivalent term in Sami for the concept of intellectual disability. They sometimes use terms like *bazahallan* or *doimmaheattigun*. These terms are metaphorical and refer to a person that does not walk with the same rhythm as others. Consequently, when in an interview speaking about *bazahallan*, this is not necessarily equivalent to term intellectual disability. Dealing with such issues during interviews demand a strong focus by the researcher's to get the informant to elaborate on their understanding. Further, the research approach opened up for an in-depth understanding of Sami culture and background, which is a prerequisite for increasing the researcher's knowledge regarding for example terms like these.

#### Awareness of Sami history, culture and communication makes better interviews

Some informants described how they perceived Norwegians to be quite direct in their language, in contrast to the Sami, who often have a more indirect way of expressing themselves. Use of metaphors and indirect language made our use of an interview guide difficult. We as researchers had to focus less on our interview guide and be more open to follow the conversation rather than being pushy with our agenda. Hence, we did not always get answers to all of our pre-prepared questions. Instead, we often got a deeper understanding of the Sami culture and people, and how the assimilation process marks individuals, families and communities even today (51). For example, we learned not to begin our small talk with questions about whether or not they could speak Sami, or if they had a herd of reindeer. One might argue that this is an ethnocentric assumption because these types of questions perpetuate stereotypes created and maintained by outsiders. However, some of the participants themselves pointed out to us that they found these types of small talk questions offensive because they were connected to shame and/or conflicts inside their family and/or inside the Sami community (7). Hence, as researchers we have a responsibility to be culturally sensitive to how the impact of assimilation on the indigenous Sami people and the relationship between Sami and non-Sami people might influence our informants and their families even today. And, in turn, influence how researchers in the north have to be sensitive to this. We have to understand the significant cultural differences between indigenous Sami people and the majority Norwegian community in terms of spiritualty, narratives, thinking, beliefs, values, etc. And also recognize that Sami society is extremely diverse; although there may be similarities, there is not one Sami indigenous culture, but many subcultures, within the Sami society. In addition, some elements of the Sami culture and identity are common with Norwegian culture and identity. All this is a part of what we define as being culturally sensitive to include indigenous perspectives in all steps of the research agenda, which fundamentally changes the way we approach and do research (36).

In addition, traditional Sami people do not easily talk about their diseases because a perception exists that talking about one's weaknesses might make things worse (52). This perception is based on the Sami concept of how human beings are inflicted with disease and suffering; illness was (and, by some, still is) considered punishment for wrongdoing. In the pre-Christian Sami religion, there are accounts of illness being regarded as a consequence of evil forces seeking to take the sick person's life (or soul), and that someone had “inflicted evil upon them” (53). Accordingly, in the conversation with our informants we focused on their strength and how they coped with their challenges, rather than focusing on their diseases or impairments.

### Analysis and accountability

#### Discussing findings with Sami people gives a deeper understanding

Even though some of our research group members had a Sami background, the researchers were all educated and trained within a Western research framework. Thus, it was important to us to include the Sami people's own framework of knowledge in the analysis process to avoid showing disrespect and causing further harm to Sami people. This inclusion involved presenting and discussing how to understand the findings with representatives from the Sami community and the disability field at some of the Sami language and cultural centres. Furthermore, we brought the representatives interpretation back to the research group and considered it in the further analysing process. Among these representatives were also some of the participants from the interviews. This sort of involvement of community members in the analysis and interpretation of the findings is recommended by Chilisa (24). Furthermore, by contributing in the analysis process, we also sought to balance the power in the process of knowledge production. When Sami (and disabled) people discuss the findings within their traditional knowledge framework, this can contribute to building a bridge between traditional Sami knowledge and traditional research-based knowledge. Our plan is to attempt this sort of bridge building in the dissemination part of the study as well, involving Sami people and people experiencing disability in this process, for example, through seminars with practitioners and politicians, and further cooperation with the Sami language and culture centres and meetings here.

## Conclusion

In this article, we identified a number of ethical and methodological issues in research of disability among Sami people in Norway. We hope that we have conveyed the message that disability research within ethnic minorities like Sami people raises more ethical and methodological challenges than research with people with majority background not experiencing disability. In relation to conducting research with Sami people experiencing disability, researchers need to have knowledge about Sami culture and history. This is to avoid the pitfalls throughout the whole research process. Due to specific situational contexts, spontaneous alterations might be needed both when entering the field, during the data collection and in the analysing and reporting process. Following the existing guidelines might not be enough to attend to our moral responsibility as researchers. To ensure that our research does not have a stigmatizing and disempowering effect on the participants, it is urgent to be aware of possible ethical and methodological issues and the need for continuous changes emerging from the planning until the fulfilment of studies on disability among Sami people.

## Footnotes

1 Harstad University College merged with University of Tromsø – The Arctic University of Norway on 1 January 2016, and is now a part of University of Tromsø – The Arctic University of Norway.
